# Use of infrared thermography for the assessment of free flap perforators in autologous breast reconstruction: A systematic review

**DOI:** 10.1016/j.jpra.2019.11.006

**Published:** 2019-12-03

**Authors:** Orla Hennessy, Shirley M. Potter

**Affiliations:** Department of Plastic Surgery, Galway University Hospital, Galway, Ireland

**Keywords:** Infrared thermography, Breast reconstruction, DIEP dynamic perforator, Mapping location

## Abstract

Perforator-based flaps have in recent years become the mainstay of autologous breast reconstruction practice. Imaging modalities ranging from Doppler ultrasound to CT angiography demonstrate varying utility in the preoperative identification and localisation of perforators. Despite these available radiological investigations, finding and quantitatively assessing perforators remain a time-consuming and tedious process that is often complicated by a number of factors including variable anatomy prior surgery and body habitus. Thermographic imaging shows promise as a novel modality for preoperative localisation of perforator vessels. This review summarises the currently available evidence for its application in perforator mapping for abdominal-based autologous breast reconstruction. We discuss the development of the technology over the years, its current use, its advantages and how it may impact on reconstructive breast surgery.

## Introduction

Since their introduction in 1989 by Koshima and Soeda,^1^ perforator-based flaps have dramatically grown in popularity. They have gained mainstream following in all areas of microsurgery, in particular reconstructive breast surgery. Currently, abdominal perforator-based flaps based on the deep inferior epigastric artery (DIEA) system are accepted as the gold standard autologous reconstructive option for breast cancer patients.^2^ Such DIEA perforating vessels show high anatomical variability in terms of location, course and size. Preoperative assessment of these perforator vessels provides a road-map to guide decision-making during flap raising and has undoubtedly contributed to a gradual refinement in the operative techniques used. Currently, Doppler ultrasonography (US), computed tomography angiography (CTA) and to a lesser degree magnetic resonance angiography (MRA) have become the mainstay objective assessment modalities for preoperative perforator planning. Their use in flap planning has been shown to reduce operating time, increase flap survival and reduced complications.^3^, ^4^, ^5^ Although all are effective in mapping perforators, they suffer from disadvantages such as lack of easy portability, requirement for specially trained operators, exposure to ionising radiation and the use of intravenous contrast medium, as well as high costs and delays in acquiring and reviewing imaging studies.^6^

Infrared thermography (IRT) is a relatively old concept, which has recently been applied to the imaging of vessels. In 1800, while measuring the temperature of light, Sir William Herschel discovered “infrared” radiation.^7^ In 1929 Kalman Tihanyi invented a camera based on the concept that all objects emit a “heat signal” in the form of infrared radiation.^8^ An infrared thermographic camera detects this radiation in the same way that a normal camera detects light,^8^ and generates visible heat maps in the same way photographs are created. The flow in perforator vessels emits a detectable heat signal, which can be recognised by IRT allowing localisation.^9^

In recent years, IRT shows great promise as a modality for preoperative localisation of perforator vessels prior to free flap surgery. Thermography is an inexpensive, portable, non-invasive imaging modality. In particular the Forward Looking Infrared (FLIR) ONE thermographic camera is a smartphone device that is portable and convenient to use. Thermography has been shown to provide objective data on perforator location.^10^ Thermography has also previously been demonstrated to be able to identify differential rewarming rates between perforators,^11^^,^^12^ which can aid in identifying interperforator anastomoses and therefore can be used to identify more favourable axes along which to design perforator flaps. This provides the reconstructive surgeon with information not previously available, and may be used to best advantage when planning perforator flaps comprising multiple perforator angiosomes.

In recent years, there have been a significant number of publications describing the use of thermal imaging in the preoperative planning as well as intraoperative and postoperative monitoring of flap perfusion, particularly in the field of reconstructive breast surgery. Herein, we perform a review of the current evidence for the use of thermal imaging in perforator mapping prior to abdominal-based perforator flaps and its potential role as an alternative or adjunct to current techniques used in autologous breast reconstruction.

## Methods

We performed a systematic review to evaluate the current evidence regarding the use of IRT in the assessment of abdominal perforating arteries. This was performed via an electronic and manual search. The literature identified was then critically evaluated using the 2009 Oxford Centre for Evidence-Based Medicine (OCEBM) definitions.^13^

## Search strategy

The search strategy comprised searching the online databases Medline, SCOPUS, EMBASE and Cochrane collaboration for all articles on the topic of IRT in perforator assessment published up to and including July 2018. The bibliographic references of the captured articles were examined in order to search for additional relevant citations. The search strategy was sensitive to texts and abstracts, with the keywords “thermography” and/or “infrared” and/or “perforator”.

## Inclusion criteria

A review of scientific literature was performed. Included were all prospective and retrospective studies including case reports, cohort studies, randomised control trials and clinical trials that analysed the applications of IRT in the assessment of abdominal perforators. Only clinical studies with human participants and cadavers were included. Inclusion criteria comprised all studies that used IRT to analyse abdominal perforating vessels.

## Exclusion criteria

Exclusion criteria included studies not carried out in humans, non-English language publications, studies centred only on perforators outside the abdomen and studies using alternative imaging methods such as near-infrared imaging.

Studies that passed inclusion and exclusion criteria were separated for full reading, critical appraisal and data collection. Data collected are as follows: identifying information on each study (including author and publication year), type of study, types of flap harvested, number of participants, study aim, infrared camera used and results.

## Results

**Figure 1:**
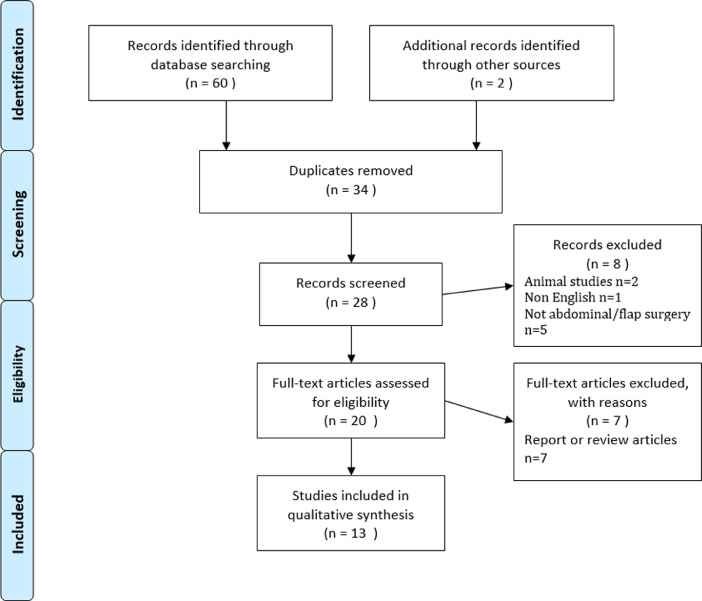
Literature review selection/exclusion of Oxford PRISMA flow diagram.

A total of 62 publications were identified using the search criteria defined above. After examination of titles and abstracts and exclusion of duplicates, a total of 13 articles were selected for in-depth reading and analysis. Excluded papers included review articles, animal studies and studies on perforators outside the abdomen as detailed in Figure 1. Two excluded studies were on animal models, seven were review articles or reports, five were not abdominal flap surgeries and one study was not in English. Table 1 provides a summary of the papers selected for further reading.

**Table 1:** Summary table of papers included in the review.

Author	Year and Journal	Participants/operation	Clinical outcome	Infrared camera used	OCEBM level of evidence
Theuvenet et al.	1986 Scandinavian Journal of Plastic and Reconstructive Surgery	4 Cadavers + 6 volunteers	Not applicable	Not described	4
Itoh et al.	1994 Annals of Plastic Surgery	12 volunteers, 2 case reports 1paraumbilical, 1 latissimus dorsi flap	Not applicable	Fujitsu Infra Eye 180	2
Salmi et al.	1995 Annals of Plastic Surgery	8 TRAM flaps	No flap failure or partial loss reported	Inframetrics 600	4
Zetterman et al.	1999 European Journal of Plastic Surgery	16 volunteers	Not applicable	Inframetrics 600	4
de Weerd et al.	2006 Annals of Plastic Surgery	7 DIEP flaps + 3 SIEA flaps	Flap reperfusion in all cases, long-term flap outcome not reported	Nikon Laird S270	3
Kalra et al.	2007 Journal of Plastic Aesthetic and Reconstructive Surgery	2 DIEP flaps	No flap failure or partial loss	NEC Thermo Tracer TH7102MV	5
de Weerd et al.	2009 Annals of Plastic Surgery	23 DIEP flaps	No flap failure Partial loss in 3 flaps (<5%)	Nikon Laird S270;FLIR Therma Cam S65 HS	3
Tenorio et al.	2011 Annals of Plastic Surgery	10 DIEP flaps	No flap failure or partial loss	BioScan IR system	2
Whitaker et al.	2011 Journal of Plastic Aesthetic and Reconstructive Surgery	1 Bilateral DIEP	No flap failure or partial loss	Not described	5
Sheena et al.	2013 Plastic and Reconstructive Surgery	20 Volunteers	Not applicable	FLIR SC660	3
Chubb et al.	2013 Plastic and Reconstructive Surgery	10 Patients	Not applicable	NEC Thermo Tracer	3
Hardwicke et al.	2016 Plastic and Reconstructive Surgery	10 Volunteers	Not applicable	FLIR ONE	3
Weum et al.	2016 BMC Medical Imaging	25 DIEP flaps	1/25 flaps failed (unrelated cause) No partial loss	FLIRThermaCAM S65 HS	2

## Included publications

### Early studies

The earliest study revealed by this review was by Theuvenet et al.^14^ In 1986 they carried out some of the first cadaver work in this area. They argued that due to individual variation in the quality and location of perforating arteries, flap selection based on anatomical landmarks alone was inadequate and a niche existed for a non-invasive method of identifying these vessels. They called this method “thermographic assessment of perforating arteries” (TAPA).

They used an early version of an infrared camera to evaluate areas commonly harvested for free flaps, using warmed saline flushed through vessels to mimic blood flow. The flaps were then surgically dissected, and 31 out of 36 perforators that were detected by infrared imaging were located and confirmed. Following this, 16 living volunteers had images taken and perforators analysed after cooling, exsanguination and the use of tourniquet following a standardised protocol (Table 2). After release of the tourniquet and during temperature normalisation, hot spots were detected with thermography, showing early utility of the technology in clinical practice.

**Table 2:** Adapted from Theuvenet et al.: example protocol for thermographic imaging of live subjects based on the anatomical area being imaged.

Trunk
(1) Exclude presence of false hotspots (tumors, inflammation).
(2) Seal off the borders of the area with adhesive drapes.
(3) Dry a drum filled with cold water and roll over the area for 5–7 min putting light pressure on the skin. Alternatively a bag of saline from the fridge can be used.
(4) Set the black level of the thermograph to a consistent temperature, e.g., 15 °C.
(5) Terminate cooling, take thermographic images during re warming at set intervals.

In 1995, Salmi et al.^15^ performed a study on transverse rectus abdominis muscle (TRAM) flaps in eight patients using thermography to map perforators pre-, intra- and postoperatively. Similar to previous studies, they again showed that thermography could be used to map perforators, and had advantages over other methods used at the time including Doppler ultrasound and MRI. This study is notable as it did not include a thermal challenge, but still found utility for IRT in the preoperative phase.

### Expansion of the temperature challenge concept

In 1995, Itoh and Arai^16^ went on to describe a “recovery enhanced” method of IRT with the use of a thermal challenge for the assessment of cutaneous perforators for the purpose of designing perforator-based flaps. They demonstrated the use of this method to locate perforators in 12 volunteers with confirmation by Doppler ultrasound. They discussed the variability in anatomical location of perforators observed, and the utility of digital thermograpy in localising these vessels.

Zetterman et al.^17^ in 1998 further explored the effect of temperature changes on the perforating vessels of the abdomen. They examined the abdominal perforators of 16 women after both warming and cooling. They showed that hotspots on thermal imaging disappeared with cooling, and reappeared with warming and also showed that longer cooling periods were associated with better visibility of perforators.

### Exploring utility in planning perforator flaps

In a 2006 paper, De Weerd et al. incorporate the use of IRT specifically for perforator assessment and flap selection, and also use it post anastomosis to provide information on completed flap perfusion. Ten patients scheduled for deep inferior epigastric perforator (DIEP) or superficial inferior epigastric artery (SIEA) flap reconstruction had IRT images taken pre-operatively at room temperature as well as following a cold thermal challenge. During the operation, sequences of digital IRT images were taken before and after clamp release, post anastomosis in four cases and post anastomosis and a cold challenge in three cases. The group found hot spots on thermography-matched Doppler ultrasound findings and concluded that “the anatomic structure related to a hot spot must be a perforator”. They demonstrated that IRT could be used to detect successful arterial inflow as well as distinguish partially and fully obstructed inflow, allowing for the intraoperative detection of sub-optimal flap reperfusion. This can be due to causes such as kinking, thrombosis or damage to the perforator, and early detection allows for correction.

In 2007, Kalra et al.^18^ conducted a pilot study on two patients who were scheduled for elective breast reconstruction with a DIEP flap. They divided the flaps into half for transfer and then an experimental half. In this half they performed sequential ligation of the major perforators and conducted thermal imaging on each and created histograms based on thermal data. They postulated that thermography was able to measure the strength of perfusion; however, as the experimental flaps were not used, there was no clinical measure regarding the outcome with the selection of perforators deemed to be “dominant”.

This was followed in 2009, by another significant contribution from de Weerd et al.^19^ building on previous work, they studied the use of IRT in the preoperative selection of perforating vessels and the planning of DIEP flaps. Twenty-seven patients undergoing DIEP (23) or SIEA (4) flap harvest were recruited. IRT images were taken before and after a cold challenge. The first hotspots to appear were checked with handheld Doppler. Eight of the patients also had multi-detector CT images recorded. The study found that the speed with which the hotspot appeared correlated with the volume of Doppler sound. Perforators were selected based on the speed and pattern of hotspot appearance as well as Doppler volume.

In the eight patients who had a CT scan, the perforators highlighted by thermographic examination related to an easily visible perforator on imaging. The authors suggest that the perforators associated with faster rewarming, and thus earlier and larger hotspots, are capable of higher blood flow, suggesting an area of improved vascularity. All DIEP flaps survived, with only partial salvageable flap loss in three cases. Thus, the perforators selected using thermography were successfully used.

In 2011, Tenorio et al.^20^ compared infrared imaging without a thermal challenge with handheld Doppler in 10 DIEP flaps. This study was strengthened by the addition of analysis of surgical dissection to confirm perforator location, providing a high reference standard. Perforator location matched within 0–15 mm in 67% of patients. They found that rather than one technique necessarily replacing the other, Doppler located perforators in the deeper level, whereas thermography localised them more superficially just under the skin surface. As such, they found the techniques complementary, and showed that a cold challenge may not be necessary.

### Further comparisons to the current “gold standard”

In 2012,^21^ Whitaker et al. performed a comparison of IRT with CT angiography – the gold standard of vascular imaging. The study only included one case, a planned bilateral DIEP reconstruction. Preoperative CTA located one suitable medial right-sided perforator. Infrared imaging similarly showed one right-sided hotspot, illustrating good correlation between the two methods. As a result it was decided to perform a unilateral reconstruction. This case illustrated how pre-operative perforator assessment can aid surgical decision-making.

In 2013, Chubb et al.^11^ again compared IRT to CTA. They showed that perforators larger than 1 mm on CTA were accurately localised using IRT. They also analysed the pattern of rewarming in inter-perforator zones. This can vary depending on whether these zones are connected by true or choke anastomoses. They postulate that infrared imaging can help identify the better-perfused areas, and thus those supplied by true anastomoses. This should increase flap quality, and as such will assist surgical decision-making.

In the most recent study, Weum et al.^22^ compared pre-operative perforator mapping by IRT with handheld Doppler and CTA. Surgical outcome was the major endpoint evaluated. In 25 women undergoing DIEP reconstruction, thermal images were taken pre and post a cold challenge and analysed for the pattern and rate of hotspot rewarming. Earlier appearing hotspots were associated with clear perforators on CTA and correlated with subjective volume assessment on Doppler. In all cases, the selected perforators were identified intra-operatively. Of the 25 flaps, 24 survived with the cause of flap failure being unrelated to perforator selection. Thus, IRT selected a suitable perforator for reconstruction in all 25 cases.

### Use in the modern plastics theatre

**Figure 2:**
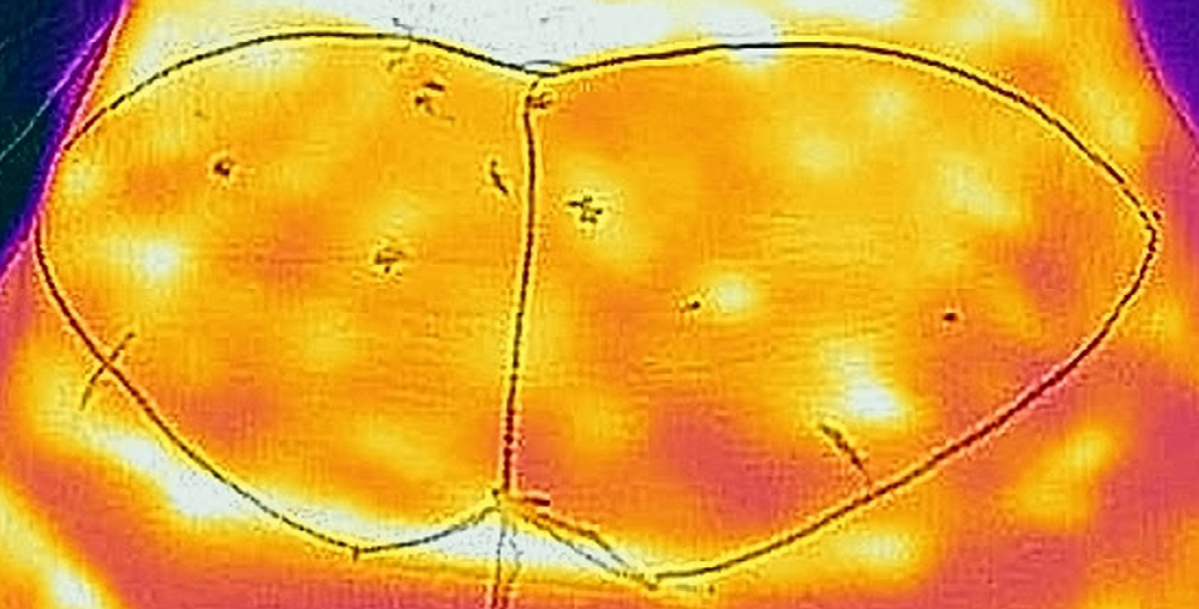
Representative abdominal thermogram recorded using the FLIR One smartphone thermal camera. Brighter colours represent higher temperatures or “hotspots” that indicate the site of DIEA perforating vessels.

In a 2013 study, Sheena et al.^23^ assess whether handheld thermal imaging devices were as effective at locating cutaneous perforators as conventional handheld Doppler ultrasound. Twenty male participants had their abdomen, sacrum and anterolateral thighs assessed for cutaneous perforators by thermal imaging. Perforators were identified and then checked against handheld ultrasound devices. In total, handheld Doppler confirmed 97% of 757 hotspots on thermal imaging to be perforators. Figure 2 gives a representative example of an abdominal thermogram.

In 2016,^24^ Hardwicke et al. conducted a similar study using smartphone thermal imaging, an even more portable and affordable version of the technology. They used the FLIR One smartphone compatible infrared imaging camera, which currently retails at approximately 200USD. In this proof-of-concept study, 10 healthy volunteers had thermographic images captured of the abdomen and lateral thigh. Perforators were signified by hotspots on thermal imaging and confirmed by Doppler ultrasound. They found that smaller thermal imaging devices, while lacking the same levels of resolution as larger cameras, readily identified perforator hotspots. These devices are simpler to use with a point and shoot style technology and an integrated app available on both android and apple. As such, they potentially provide a low cost, but effective alternative to larger devices, and their role is likely to complement perforator mapping available with CTA.

## Discussion

Selection of a suitable perforator is an important aspect of the preoperative planning of free flap procedures.^19^ Although it is possible to do this intra-operatively, variability in location and size of perforating vessels makes this difficult.^25^ Preoperative mapping allows the flap to be designed around a dominant perforator ahead of time, and serves to decrease operation length.^9^ This can be done using a number of different methods including MRI, CTA and Doppler ultrasound; however, each of these is with their own disadvantages.

Doppler ultrasound is operator-dependent with low sensitivity for the identification of perforators and high inter-user variation. MRI, ICG angiography and CTA are expensive, require formal training to use and interpret and involve the use of contrast and/or ionising radiation. MRI and CTA provide no information on perforator quality and cannot be used for intra-operative or post-operative monitoring.^26^ Additionally, MR and CT are time-consuming and dependent on scanner availability and waiting lists and require radiology input for interpretation and reporting purposes. Conversely, IRT can be performed and interpreted by the operating surgeon either preoperatively or in a preliminary clinical setting and takes only 5 min to perform once people are initially trained. As such, IRT presents a quicker, less invasive method of assessing skin perfusion, without the risks associated with ionising radiation and contrast injection.^25^

**Figure 3:**
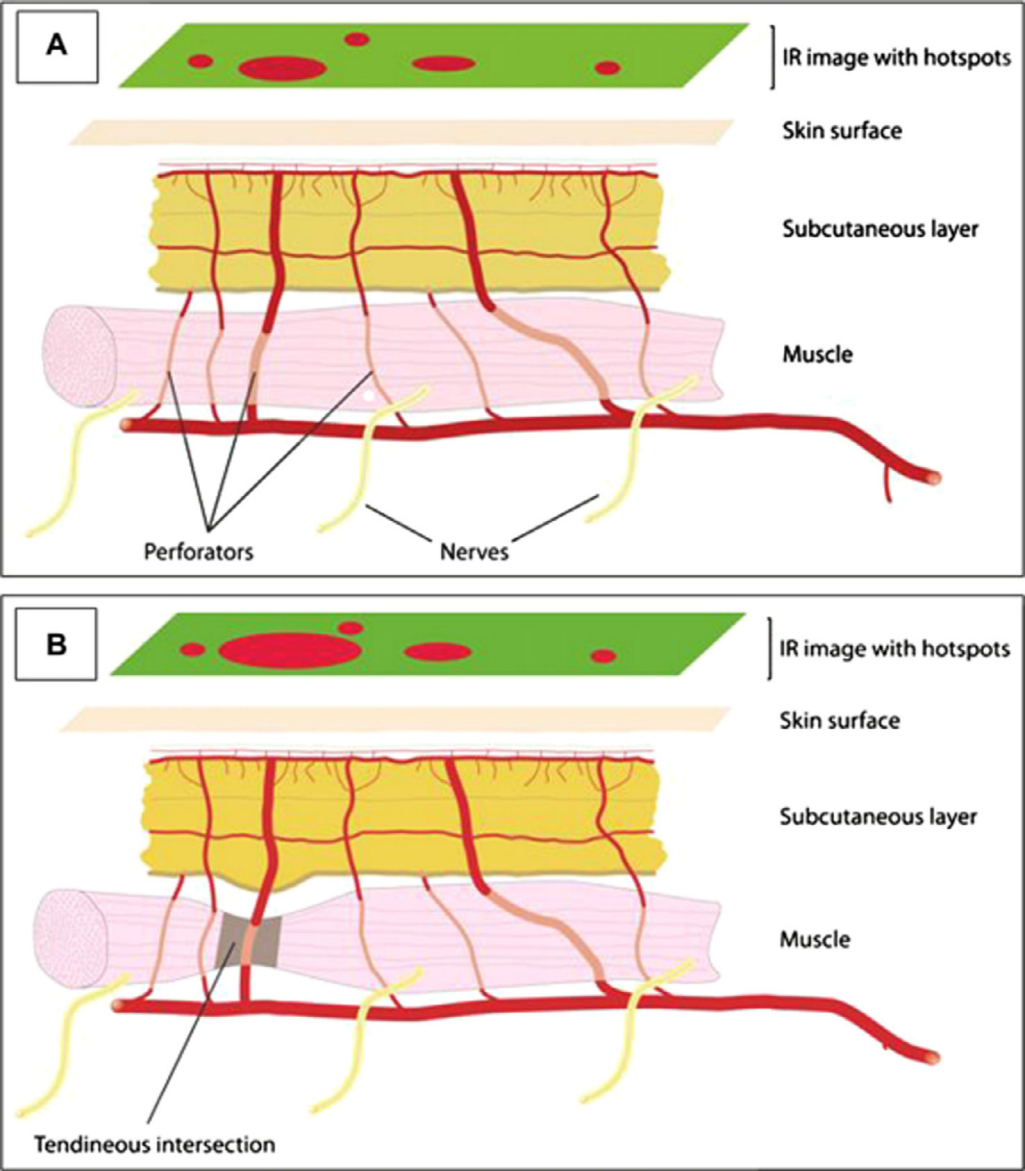
Direct perforators with a short intramuscular course may be associated with a bright hotspot (A). Direct perforators at the tendinous intersection have a short intramuscular course and have a calibre larger than the average, and show up as bright hot spots (B). Reproduced from de Weerd et al.^9^ with kind permission of the authors.

In terms of surgical planning, the least difficult dissection is reported for perforators with a perpendicular penetration pattern through the fascia and a short intramuscular course. Perforators that are located at tendinous intersections have these characteristics and are reported to be larger than average, likely increasing the quality of the perforator.^2^ These perforators are easily identified with IRT as the short straight course of the perforator facilitates rapid rewarming of the skin causing a bright, early hotspot (Figure 3A and B). de Weerd et al. concisely illustrated this in a 2011 report.^27^ They illustrate the advantage of infrared imaging in allowing the user to assess perforator quality, ease of access and location. Overall, they concluded that “information obtained from infrared thermography examinations can be of great value to the plastic surgeon in perforator flap surgery”.

## Limitations

As such, IRT can be of great value to the plastic surgeon. However, the technology is not without limitations. First, IRT only provides information on perforator location as opposed to morphology and physiology. Second, any anatomical information provided is in the form of a superficial, two-dimensional map. This is in contrast to 3D mapping available with CTA and MRA. As such, it remains important that the surgeon retains in-depth knowledge of local vascular and flap anatomy when interpreting these images. Additionally, at the present time IRT is not widely available, and few people are trained in its use, though this is likely to change as the technology becomes increasingly affordable. Smartphone-based IRT in particular holds promise as an easily accessible, inexpensive version of the technology, though will require regulation in regard to compliance with data protection principles.

It is also important to note that in addition to providing a two-dimensional map, IRT will only provide information to a certain depth. Thermographic imaging is dependent on surface skin temperatures, which is influenced by structures up to a depth of approximately 2 cm.^28^ This means that perforators terminate further from the skin surface in subcutaneous tissues may not be detected, and therefore be overlooked in flap planning via IRT. These perforators are better identified by CTA.^22^

Another limitation associated with thermographic imaging is controlling for temperature interference from the surrounding skin. This is particularly important when using lower resolution (and generally more affordable) devices. Skin temperature can fluctuate by as much as 8° depending on factors including clothing, room temperature and humidity.^29^ In dynamic IRT, allowing a period of acclimatisation to a consistent room temperature, followed by cooling to a set temperature helps to minimise this interference.^9^ Cooling and imaging during rewarming also help to more accurately identify hotspots and improve image resolution.^17^ While initial concerns were raised regarding cooling resulting in vasoconstriction prior to flap harvest, gentle cooling within the physiologic range results in reperfusion within 5 min and is deemed safe.^6^

Finally, it is important to note that the studies described are small series, with the largest only including up to 25 patients. Across the trials there is also little homogeneity in terms of camera/software used, outcomes measured and reference standard (Doppler vs. surgical dissection vs. CT/MRA), which makes it difficult to draw any composite conclusions from the cohort as a whole or perform a meta-analysis of the available data. Despite this, the overwhelmingly positive outcomes of each individual trial, over a period of almost 30 years, illustrate the utility of the technology and highlight an overall lack of negative outcomes associated with its use. This use is also not isolated to abdominal perforator flaps, and has also shown utility in planning anterolateral thigh (ALT) among other perforator flaps.^30^ In order to solidify thermal imaging's place in the plastic surgery operating theatre, there is a need for larger, controlled trials with defined outcomes offering direct comparison to current gold standards (CTA, MRA). The favourable outcomes of the studies outlined above, coupled with continued technological advances, are likely to encourage further research in this area.

## Conclusion

Thus, IRT has evolved over time to become widely applicable in the medical sphere. Competition is driving the market, as numerous companies currently have patents pending or granted for variations in the current technology. As lower-cost, easy-to-use options become more and more available, this will only serve to further encourage use of the technology and research surrounding it.

In flap planning and harvesting, IRT allows for a non-ionising, non-invasive method of assessing perforator location and quality and thus assists in surgical decision-making in real time. This may allow for higher quality and more efficient operations and potentially improve outcomes for patients; however, more experience and research with this technology is required. Despite its historical roots, the fact that this technique is relatively novel in the surgical sphere means that much more research will be undertaken in the years to come, and IRT could become a standard component of free flap protocols. Again, this is increasingly likely as low-cost, easy-to-use options become available. Future studies should include randomised controlled trials assessing whether short- and long-term flap outcomes are improved with IRT.

## Declaration of Competing Interest

None.
